# A novel conditional ZsGreen-expressing transgenic reporter rat strain for validating Cre recombinase expression

**DOI:** 10.1038/s41598-019-49783-w

**Published:** 2019-09-16

**Authors:** Elizabeth C. Bryda, Hongsheng Men, Daniel J. Davis, Anagha S. Bock, Mary L. Shaw, Kari L. Chesney, Miriam A. Hankins

**Affiliations:** 1 Rat Resource and Research Center, University of Missouri, Columbia, Missouri United States of America; 2 Department of Veterinary Pathobiology, College of Veterinary Medicine, University of Missouri, Columbia, Missouri United States of America; 3 Animal Modeling Core, University of Missouri, Columbia, Missouri United States of America; 4 Comparative Medicine Program, College of Veterinary Medicine, University of Missouri, Columbia, Missouri United States of America

**Keywords:** Genetic engineering, Gene expression

## Abstract

The Cre/*lox*P recombination system has revolutionized the ability to genetically manipulate animal genomes in order to conditionally control gene expression. With recent advances in genome editing, barriers to manipulating the rat genome have been overcome and it is now possible to generate new rat strains (Cre drivers) in which Cre recombinase expression is carefully controlled temporally and/or spatially. However, the ability to evaluate and characterize these Cre driver strains is limited by the availability of reliable reporter rat strains. Here, we describe the generation and characterization of a new transgenic rat strain in which conditional expression of the ZsGreen fluorescent protein gene requires the presence of exogenous Cre recombinase. Breeding Cre-expressing rat strains to this stable ZsGreen reporter strain provides an ideal method for validating new rat Cre driver lines and will greatly accelerate the characterization pipeline.

## Introduction

The Cre/*lox*P recombination system has revolutionized the ability to genetically manipulate animal genomes in order to spatially and temporally control gene expression^1^. This has resulted in the generation of a large number of mouse strains, often called “Cre drivers”, in which Cre recombinase expression is carefully controlled. While this technology has been used in the mouse for decades, the previous lack of genetic tools to efficiently engineer the rat genome has led to a paucity of rat Cre drivers. With recent advances in rat embryonic stem cell technology and general genome editing, including methods involving CRISPR/Cas9, barriers to manipulating the rat genome have been overcome and there is an increasing interest in using rat models instead of mouse models. This creates a new need to generate Cre driver lines in rats. An ideal method for validating new Cre driver lines involves breeding to a conditional reporter strain and assessing Cre recombinase expression based on resulting expression of a fluorescent protein gene such as green fluorescent protein (GFP). However, the rat reporter strains described in the literature to date are limited^2–4^ or in at least one case, the transgene has been silenced and the strain no longer expresses the reporter gene even in the presence of Cre recombinase (^5^ and unpublished data, personal communications). In some cases, access to reporter strains may be hindered. For example, the W-Tg(CAG-DsRed2/GFP)1Jmsk and W-Tg(CAG-DsRed2/GFP)15Jmsk strains are DsRed2/GFP double-reporter transgenic rats with expression driven by a CAG promoter. DsRed2 expression is replaced with GFP expression after Cre recombinase-mediated excision^4^. The strains are available as cryopreserved material through the National BioResource Project for the Rat in Japan (http://www.anim.med.kyoto-u.ac.jp/NBR) and the Rat Resource and Research Center in the US (http://www.rrrc.us). The strains would need to be cryorecovered in order for an investigator to use them, resulting in a delay in the availability of live animals. Another reporter strain is SD.Rosa26(tm-imCherry)-GC/ILAS^2^. These rats have ubiquitous expression of EGFP unless Cre recombinase is present. Cre-mediated recombination results in deletion of the EGFP gene with subsequent expression of mCherry instead^2^. This strain is available currently as live animals at the Rat Resource in China (www.ratresource.com) and represents one of the few well-characterized rat reporter strains currently available as live animals. The need for reliable reporter rat strains with stable expression of a variety of fluorescent proteins that are readily accessible to the biomedical community is critical. Here we describe a new strain (F344-Tg(CAG-*lox*P-STOP-*lox*P-ZsGreen) that has consistent and specific expression of ZsGreen in the appropriate tissues as dictated by the expression pattern of Cre recombinase. A benefit of ZsGreen is that it is >8-fold more intense than EGFP^6^. We present data that shows appropriate ZsGreen expression when the rats were bred to both a ubiquitously expressing-Cre strain, Wistar-Tg(CAG-Ncre)81Jmsk^4^, and a cell-type specific-Cre expressing strain, LE-Tg(Chat-Cre)5.1Deis^7^. Importantly, we show that the ZsGreen transgene continues to exhibit stable expression in the presence of Cre recombinase even in reporter animals multiple generations removed from the original founder animal, demonstrating that transgene silencing has not occurred over time as has been seen in other transgenic reporter strains.

## Results

### Generation of conditional ZsGreen-expressing rats

**Figure 1:**
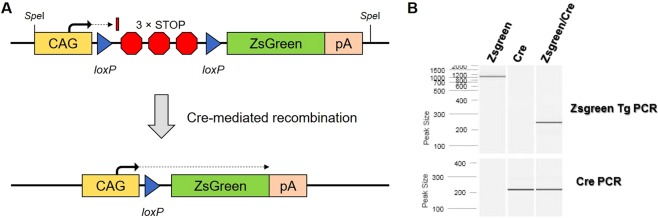
ZsGreen transgene. (**A**) The transgene construct contains a chicken β-actin promoter with a CMV enhancer element followed by a chimeric intron. The floxed-STOP region contains *lox*P sites flanking 3 tandem SV40 polyadenylation signal sequences. Downstream of the floxed-STOP cassette is the ZsGreen gene sequence (originally derived from a *Zoanthus* sp. reef coral) followed by a bovine growth hormone polyadenylation signal. Upon Cre recombinase-mediated recombination, the *lox*P sites are recombined resulting in the excision of the 3 tandem SV40 polyadenylation signals. This event allows expression of the ZsGreen gene. (**B**) PCR amplification to demonstrate removal of the floxed STOP region from the ZsGreen-containing transgene. Lane 1: ZsGreen transgenic animal; Lane 2: NCre transgenic animal; Lane 3: double transgenic animal. Upper panel: results of amplification with primers flanking the floxed STOP region. Lower panel: results of amplification with primers that detect the Cre recombinase gene. A molecular size standard is shown to the left of the image. For reference, the uncropped image is included as Fig. S1.

Reporter rats were generated by random transgenesis through microinjection into F344/NHsd zygotes. The incorporated transgene contains a ZsGreen gene downstream of a floxed STOP cassette (Fig. 1A). In the presence of Cre recombinase, *lox*P site-specific excision of the STOP cassette occurs (Figs. 1B and S1). This results in expression of the ZsGreen gene driven by the ubiquitously active chicken β-actin promoter coupled with the CMV early enhancer (CAG). Two independent male founders, confirmed by PCR analysis to carry the transgene, were subsequently bred to F344/NHsd females to establish two independent lines, F344-Tg(CAG-*lox*P-STOP-*lox*P-ZsGreen)551Bryd (RRRC# 795) and F344-Tg(CAG-*lox*P-STOP-*lox*P-ZsGreen)561Bryd (RRRC# 797). Colonies were established by continual backcrossing of hemizygous transgene positive animals to F344/NHsd animals. The transgene has been stably maintained for 4 and >8 generations for Lines 551 and 561 respectively (data not shown).

### Copy number and integration site analysis

Each line had integration of the transgene at a single insertion site. Interestingly, targeted locus amplification (TLA)^8^, indicated that the integration location for both lines was on Chromosome 2 (Fig. S2). For line 551, the integration site was at Chr2:258601453 and for line 561, the integration site was at Chr2:209721072 based on mapping to the rat genome, version Rattus_norvegicus.Rnor_5.0^9^. While exact copy number cannot be determined reliably using TLA, copy number estimates in a hemizygous animal were >20 for line 551 and 3–10 for line 561. To more accurately assess the number of transgene copies that had integrated at the single Chr. 2 insertion site for each line, droplet digital PCR (ddPCR)^10^ was performed. Based on this analysis, hemizygous animals from line 551 carry approximately 35 integrated transgene copies and hemizygous animals from line 561 carry approximately 4 transgene copies at their respective Chr. 2 integration sites. Analysis of homozygous samples confirmed the copy number estimates. Based on ddPCR analysis, approximately 70 and 8 copies of the transgene are detected in homozygous animals from line 551 and 561 respectively (Fig. S2). Since we saw no differences in levels of expression in the studies to be described subsequently, we chose to cryopreserve line 551 and the data presented in this report shows the analysis for animals carrying the line 561 transgene. This strain continues to be maintained as a live colony at the Rat Resource and Research Center (RRRC).

### Cre-dependent ZsGreen expression

Because of the STOP cassette, ZsGreen should not be expressed in transgene positive animals. However, in the presence of Cre recombinase, *lox*P-site specific recombination removes the STOP cassette, allowing expression of ZsGreen in tissues and cell types in which the CAG promoter is active. Hemizygous rats from line 561 were mated to hemizygous rats from the Wistar-Tg(CAG-Ncre)81Jmsk strain also known as NCre rats^4^. This strain carries a transgene with the Cre recombinase gene under control of a CAG promoter and therefore expression of Cre is expected to be ubiquitous. One finding that became clear in the course of these studies was that the transgene in the NCre rats is inserted on the X Chromosome. This was based on the genotypes of offspring from a series of breeding experiments where inheritance of the transgene clearly followed expected Mendelian ratios for an X-linked transgene. Hemizygous males never transmitted the transgene to their male offspring and hemizygous females transmitted the transgene to half of their offspring regardless of sex. Because of this finding, crosses to analyze expression of ZsGreen in the presence of CAG-Cre recombinase were performed between line 561 hemizygous males and hemizygous NCre females.

**Figure 2:**
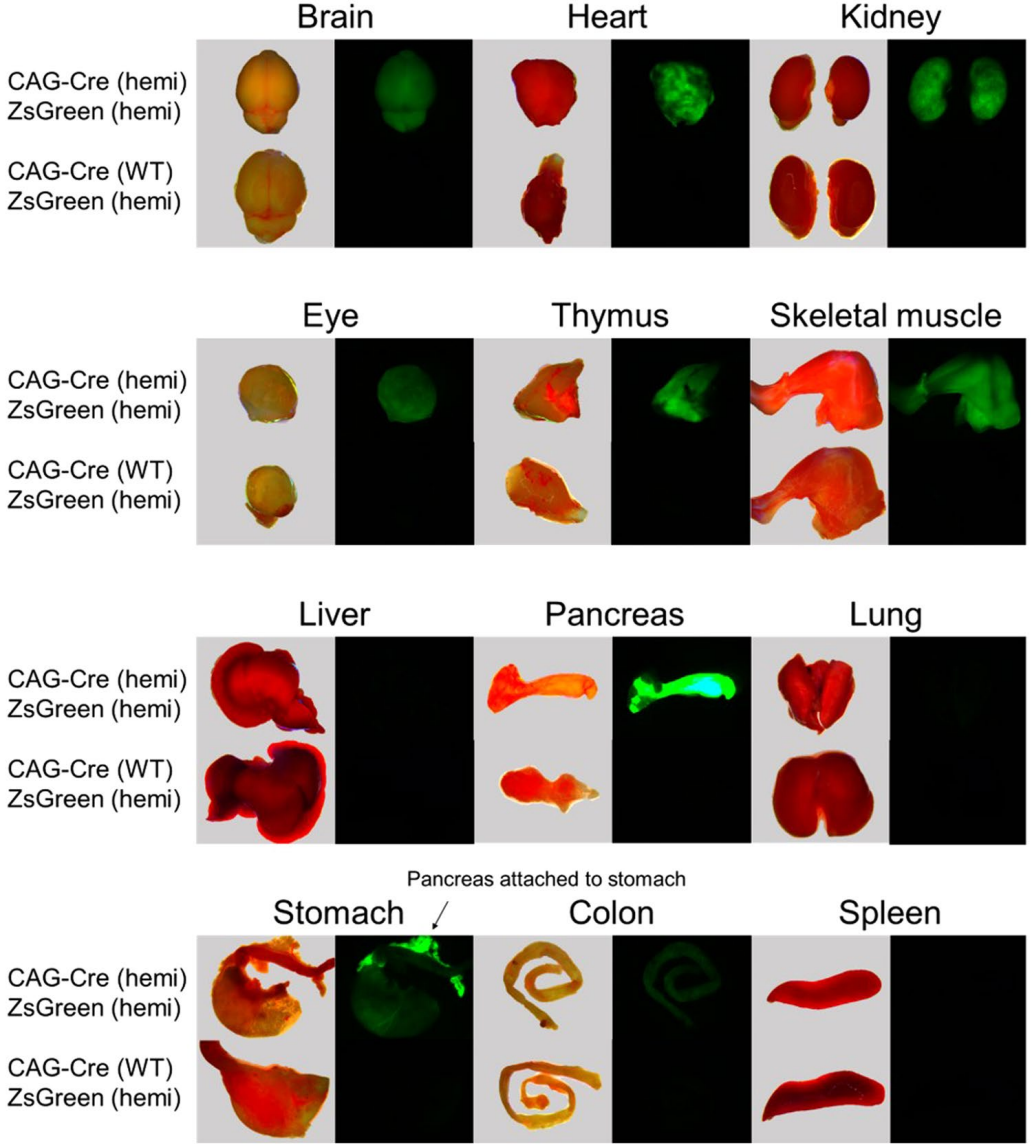
Fluorescent images of individual tissues dissected from representative 3–4 week old rats of various genotypes. Images of fresh tissue were taken immediately after euthanasia at 1X magnification using a dissection microscope under bright field (images to left). Fluorescent images were taken at 1X using 488 nm excitation with an emission filter of 503 to 563 nm (images to right). Tissues from a double hemizygous rat are shown on top and tissues from a littermate that carries only the ZsGreen transgene and not the NCre transgene are shown on the bottom.

The ubiquitous expression of ZsGreen in rat pups hemizygous for both transgenes was readily apparent even at birth by examining the pups under fluorescent light (488/503–563 nm excitation/emission wavelengths). To more globally assess ZsGreen expression in double hemizygous animals, a number of major organs were collected from 3–4 week old animals. Tissues were examined by fluorescence microscopy (Fig. 2). Table 1 summarizes the results. Similar to the expression patterns reported when NCre rats were mated to a LacZ reporter rat (SD-Tg(CAG-lacZ)541Htsu)^11^, no expression was seen in liver and spleen perhaps due to lack of or low CAG promoter activity in these tissues. While the CAG promoter is commonly used for ensuring ubiquitous and robust expression, low expression of transgenes under control of this promoter have been noted in some organs in other transgenic rat strains^12^. Differences in the level of tissue expression of the reporter genes (ZsGreen in this study, LacZ in the previously reported study^11^) were noted; for example, in our rats, no expression was seen in lung (Table 1) whereas mild expression was noted in the previous LacZ expression study^11^. These differences are most likely due to effects related to influences of the specific transgene integration sites in the two reporter strains.

**Table 1:** Expression pattern comparison in double hemizygous transgenic animals from crosses between reporter strains and NCre.

Organ/Tissue	ZsGreen X NCre	LacZ X NCre^11^
Brain	+	+
Heart	++	+++
Kidney	++	++
Eye	+	ND
Thymus	++	ND
Skeletal muscle	++	+++
Liver	−	−
Spleen	−	−
Pancreas	+++	+/−
Lung	−	+
Stomach	+	+
Colon	+/−	+++
Ovary	ND	+
Testis	ND	ND

−, negative; +/−, weakly positive; +, mildly positive; ++, moderately positive; +++, strongly positive; ND, not done.

**Figure 3:**
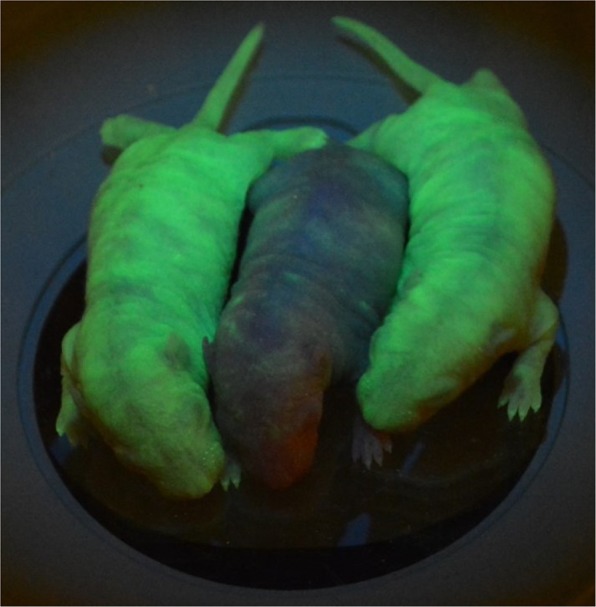
Fluorescent images of postnatal day 2 (P2) littermates from a cross between a homozygous ZsGreen line 561 male and a hemizygous NCre female. Sexes from left to right: female, male, female. Images were taken at 1X under a dissection microscope using 488 nm excitation with an emission filter of 503 to 563 nm.

It should be noted that in these crosses, it is possible to see expression of ZsGreen in offspring that do not inherit the Cre transgene. It is well documented that maternally-derived transcripts are active in the early embryo^13^. In our studies, if the ubiquitously expressed Cre transgene was carried by the female in these crosses, evidence of Cre recombinase activity (*i.e*. deletion of the STOP cassette) was detected at the DNA level and via fluorescent protein expression in offspring that carried the ZsGreen transgene but did not carry the Cre recombinase transgene (Fig. 3). We speculate that this is due to expression of maternally-derived Cre transcripts in the embryos. The same males used in these crosses were also mated to hemizygous ZsGreen transgene females. The transgene positive offspring did not have evidence of either a genetic alteration involving the transgene or expression of ZsGreen indicating that there was not some genetic change or failure of the STOP sequence in the strain. These data demonstrate that if females of the Wistar-Tg(CAG-Ncre)81Jmsk strain are used in combination with a strain carrying a floxed allele, it may be possible to mediate recombination in the offspring without the inheritance of the Cre recombinase transgene.

### Cell type-specific ZsGreen expression

**Figure 4:**
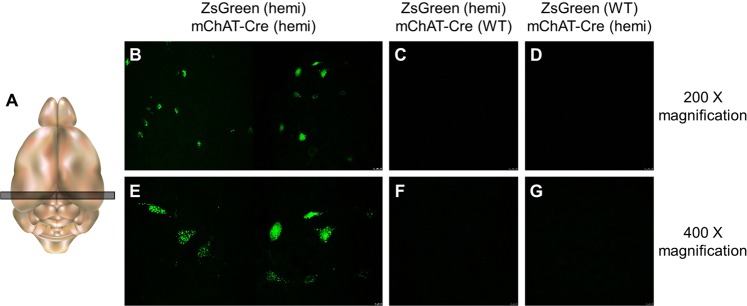
ZsGreen expression in the brains from offspring of a cross between ZsGreen transgenic X mChat-Cre-expressing animals. Representative confocal images of brain slices from three-week old rats (n = 14). (**A**) Schematic indicating location where brain slice section was taken. Images were taken at 200X magnification (**B**–**D**) and 400X magnification (**E**–**G**) under 488 nm excitation with an emission filter of 503 to 563 nm.

To examine if the ZsGreen reporter strain worked well in conjunction with a cell-type specific Cre driver strain, ZsGreen line 561 rats were mated to LE-Tg(Chat-Cre)5.1Deis rats^7^. This latter strain carries a transgene in which the Cre recombinase gene was introduced immediately before the ATG start codon of the mouse choline acetyltransferase (*Chat*) gene in BAC RP23-246B12 and therefore, Cre expression is restricted to the cholinergic neurons^7^. Analysis of brain slices from the hippocampus was performed to examine neurons of the medial septum, nucleus basalis, and nucleus accumbens. Three-week old rats showed ZsGreen expression in select neurons, presumably cholinergic neurons, in double hemizygous animals but not in animals carrying the ZsGreen transgene only or the mChat-Cre transgene only (Fig. 4). Interestingly, ZsGreen expression appeared limited to the cell body of the neurons and was quite punctate in appearance.

## Discussion

We describe the generation and characterization of a new transgenic rat strain in which conditional expression of the ZsGreen fluorescent protein gene requires the presence of exogenous Cre recombinase. Breeding Cre-expressing rat strains to this stable ZsGreen reporter strain provides an ideal method for validating new rat Cre driver lines. The utility of this particular reporter strain is in confirming that Cre recombinase is being expressed and that it is being expressed in the expected organ/tissue. Using a Cre driver strain that expresses Cre under control of the CAG promoter^4^, we showed that double transgenic animals had robust ZsGreen expression in the majority of organs examined. It should be noted that we did not see expression in lung, liver or spleen when these organs were examined grossly. This lack of expression may be due to a positional effect related to the particular insertion site of the ZsGreen transgene. However, since we did not do additional analysis either by sectioning these three organs or by using a secondary detection method, it is possible that ZsGreen was present at very low levels histologically. Once Cre lines driven by lung-, liver- and/or spleen-specific promoters known to be active in adult tissue are available to test with the ZsGreen reporter strain, it will be possible to better assess if appropriate ZsGreen expression can be seen in any cells within these organs. To test our reporter with a cell-type specific Cre driver strain, we chose a well-characterized and often used mChat-Cre rat strain^7^. The ZsGreen expression pattern in the brain of double transgenic rats was consistent with the expected pattern seen in other experiments involving specific expression in *mChat*-expressing neurons. We noted a punctate expression pattern in the cell bodies of neurons in our cross with the *Chat*-driven Cre recombinase. A review of the literature indicates that this punctate pattern has been seen in other studies involving ZsGreen expression in neurons but it is not clear if it is related to a general characteristic of ZsGreen expression or if it is cell-type specific^14–16^. This finding should be taken into consideration when interpreting expression results such that while labeling of a particular neuronal cell type is possible, it might not be possible to label all parts of the cell (*i.e*. axons, dendrites) due to the restricted localization of the ZsGreen within the cell body. Despite these caveats, the stable, strong expression of a fluorescence gene in this reporter rat line provides a reliable and powerful tool for evaluating new rat Cre-driver strains as they are being developed and characterized.

## Methods

### Generation of ZsGreen transgenic rats

All experimental procedures were approved by the University of Missouri’s Institutional Animal Care and Use Committee and were performed according to the guidelines set forth in the Guide for the Use and Care of Laboratory Animals. The pCAG-loxP-STOP-loxP-ZsGreen plasmid was a gift from Pawel Pelczar (Addgene plasmid # 51269)^17^. A linearized gel-purified *Spe*I fragment from this plasmid at a concentration of 2 ng/µL was used for the microinjections (Fig. 1). Of the 354 F344/NHsd microinjected zygotes, 210 were transferred to 8–9 week old Crl:CD(SD)(Charles River, Kingston, NY) pseudo-pregnant female recipients. A total of 22 live pups were born of which three were transgene positive. Two of these founder animals, both males, were bred to F344/NHsd females (Envigo, Indianapolis, IN) to confirm germline competency. Both were found to be germline competent and were used to establish two independent lines: 551 and 561. The lines are available from the Rat Resource and Research Center (RRRC) (www.rrrc.us) as F344-Tg(CAG-*lox*P-STOP-*lox*P-ZsGreen)551Bryd (RRRC# 795) and F344-Tg(CAG-*lox*P-STOP-*lox*P-ZsGreen)561Bryd (RRRC# 797).

### Animal crosses

Lines 551 and 561 have been maintained by backcrossing hemizygous ZsGreen transgene positive animals to F344/NHsd animals for 4 and >8 generations respectively. To demonstrate the ability to remove the floxed STOP to allow ZsGreen expression in the presence of Cre recombinase, hemizygous ZsGreen transgene positive animals were mated to hemizygous animals from either strain LE-Tg(Chat-Cre)5.1Deis (RRRC# 658)^7^ or Wistar-Tg(CAG-Ncre)81Jmsk (RRRC# 301)^4^. Both Cre-expressing strains are available from the Rat Resource and Research Center (www.rrrc.us).

### Genotyping

DNA was extracted from tail snips of 2 week old rats using the KOD Xtreme® Hot Start PCR protocol (Millipore Sigma, St. Louis, MO). Primers used and expected amplicon sizes are listed in Supplementary Table 1. PCR reactions (20 µL volume) for detecting the CAG-Cre recombinase (strain RRRC# 301) and ZsGreen (strains RRRC# 795 & 797) transgenes included 30–40 ng genomic DNA, 10 µL KOD buffer, 4 µL KOD dNTPs, 0.3 µL each of 25 µM primers, and 0.4 µL KOD polymerase. PCR reactions (20 µL volume) for detecting the mChat-Cre transgene (strain RRRC# 658) included 30–40 ng genomic DNA, 10 µL Sigma Ext-N-Amp solution and 0.3 µL each of 25 µM primers. PCR reactions (20 µL volume) for the assay to determine sex by amplification of sequences unique to the X and Y Chromosomes included 30–40 ng genomic DNA, 2 µL 10X Roche FastStart *Taq* buffer, 3.2 µL 1.25 mM Promega dNTPs, 0.3 µL each of 25 µM primers, and 0.2 µL FastStart *Taq* polymerase. Reactions were performed in 200 µL thin-walled PCR tubes in a Bio-Rad T100 thermal cycler (Hercules, CA) with thermal cycler conditions listed in Table S1. PCR amplicons were analyzed using the QIAxcel Advanced capillary electrophoresis system (Qiagen-USA, Germantown, MD) with the QIAxcel DNA Screening Kit, QX Alignment Marker 15 bp/3 kb, QX DNA Size Marker 100 bp–3 kb. The AM320 injection method with injection of 10 s at 5 kV and separation of 320 s at 6 kV was used.

### Droplet digital PCR (ddPCR)

DNA was extracted from rat tail snips using Qiagen’s DNeasy Blood and Tissue Kit, following the spin-column protocol for animal tissues (Qiagen – USA, Germantown, MD). The DNA was quantified using the Nanodrop 8000 and diluted with nuclease-free H_2_O to a working concentration of 10 ng/µL. Primers to detect the Zsgreen1 transgene were 5′-GTG TAC AAG GCC AAG TCC GT-3′ and 5′-CCA CTT CTG GTT CTT GGC GT-3′; the Zsgreen1 transgene probe was 5′-/56-FAM/TTC ATC CAG/ZEN/CAC AAG CTG AC/3IABkFQ/-3′. Primers to detect the *Ggt1* reference gene were 5′-CCA CCC CTT CCC TAC TCC TAC-3′ and 5′-GGC CAC AGA GCT GGT TGT C-3′; the *Ggt1* probe was 5′-/5HEX/CCG AGA AGC/ZEN/AGC CAC AGC CAT ACC T/3IABkFQ/-3′. Each 26 µL ddPCR reaction consisted of 13 µL BioRad ddPCR Supermix for Probes (no dUTPs), 0.5 µL of each 25 mM Zsgreen1 transgene primer, 0.5 µL of each 25 mM *Ggt1* primer, 0.25 µL of the 20 mM Zsgreen1 transgene probe, 0.25 µL of the 20 mM *Ggt1* probe, 0.25 µL of *Mse*I (5 U/µL), 7.25 µL of nuclease-free H_2_O, and 3 µL of 10 ng/µL DNA. All further pipetting was performed using Rainin LTS 200 uL filter tips to prevent destruction of droplets. Droplets were generated using the BioRad QX200 Droplet Generator, and reactions were run in a BioRad C1000 Touch Thermal Cycler with 96-Deep Well Reaction Module. Thermal cycler parameters were 1 cycle at 95 °C for 10 minutes, 40 cycles of 94 °C for 30 seconds (ramp rate of 2 °C/second) and 60 °C for 1 minute (ramp rate of 2 °C/second), and 1 cycle of 98 °C for 10 minutes. After cycling, the plate was loaded into the BioRad QX200 Droplet Reader, and BioRad QuantaSoft software, version 1.7.4.0917, was used to analyze droplet fluorescence. This data was transferred to the BioRad QuantaSoft Analysis Pro software, version 1.0.596, for data analysis.

### Transgene copy number and integration site determination

Integration sites and transgene copy number for each line were determined by targeted locus amplification (TLA)^8^. Spleen cells from 12 week old rats, a hemizygous animal for Line 551 and a homozygous animal for Line 561 were isolated using protocols provided by Cergentis (Utrecht, Netherlands). TLA was performed by Cergentis using two sets of custom primers. The Rattus_norvegicus.Rnor_5.0 genome sequence was used for mapping to determine transgene integration site.

### Tissue isolation and preparation

All animals were euthanized by carbon dioxide inhalation in accordance with the 2013 AVMA Guidelines on Euthanasia. Animals were perfused immediately with phosphate buffered saline (pH 7.4) to remove blood from tissues and then with 4% paraformaldehyde fixative to preserve tissue integrity until imaging. Whole tissues were removed and placed in sterile petri dishes on ice. Contents were removed from gastrointestinal-derived tissues prior to imaging. Tissues collected include brain, lung, heart, eye, thymus, liver, spleen, kidney, stomach, pancreas, colon, and femoral skeletal muscle. Tissues were immediately taken, on ice, for confocal microscopy imaging.

### Brain slice tissue preparation

Animals were transcardially perfused with ~50 mL of PBS followed by 4% paraformaldehyde (36 mL total). Vessels caudal to the heart were occluded with a hemostat to allow for brain-specific perfusion. The brains were then dissected carefully and post-fixed in 4% paraformaldehyde for 24 hours. After post-fixation, the brains were equilibrated to 15% sucrose until they sank to the bottom of the tube and were then stored in 30% sucrose until sectioning. Brains were cut into 40 µm thick sections (caudal to cranial) on a Microm HM 505 N cryostat (Fisher Scientific, Hampton, NH). Serial sections were stored at 4 °C in 1X PBS in 12-well plates. For analysis, sections were mounted with Prolong Gold anti-fade reagent with DAPI (Life Technologies, Eugene OR) on glass slides with coverslips.

### Microscopy and imaging

ZsGreen fluorescence in rat tails was visualized on a Nikon SMZ1500 microscope with a GFP filter. Live animals were imaged using a M165FC dissecting microscope (Leica, Solms, Germany) and DFC450 camera (Leica, Solms, Germany). Individual tissues were kept on ice and imaged immediately after dissection also using a M165FC dissecting microscope with DFC 450 camera. Confocal images of brain slice slides were obtained on a TCS SPE system equipped with a DMI 4000B confocal microscope (Leica, Solms, Germany). All fluorescent images were taken under 488 nm excitation with an emission filter of 503 to 563 nm.

## Supplementary information

Supplementary Information

## Data Availability

The rat strains described in these studies are deposited in the Rat Resource and Research Center (RRRC) and are available to the community for research use. Associated data and protocols are also available on the publically accessible RRRC web site (www.rrrc.us).
