# Supplementary material for: A novel conditional ZsGreen-expressing transgenic reporter rat strain for validating Cre recombinase expression
Source: Sci Rep. 2019 Sep 16;9:13330. doi: 10.1038/s41598-019-49783-w (PMC6746712; doi:10.1038/s41598-019-49783-w)
Supplement: Supplementary file 1 — Supplementary Information [file 41598_2019_49783_MOESM1_ESM.pdf]

A novel conditional ZsGreen-expressing transgenic reporter rat strain for validating Cre recombinase expression

Elizabeth C. Bryda[1,2,3,4*], Hongsheng Men[1,2], Daniel J. Davis[3], Anagha S. Bock[1,2], Mary L. Shaw[1,2], Kari L. Chesney[4], Miriam A. Hankins[1,2]

[1]Rat Resource and Research Center, University of Missouri, Columbia, Missouri, United States of America

[2]Department of Veterinary Pathobiology, College of Veterinary Medicine, University of Missouri, Columbia, Missouri, United States of America

[3]Animal Modeling Core, University of Missouri, Columbia, Missouri, United States of America

[4]Comparative Medicine Program, College of Veterinary Medicine, University of Missouri, Columbia, Missouri, United States of America

*Corresponding author, brydae@missouri.edu

Supplementary Information

Table S1. Genotyping Assays

Assay Name	Primers	Thermocycler Parameters	Expected Products
Cre Recombinase PCR	Forward: 5’-GCGGCATGGTGCAAGTTGAAT-3’ Reverse: 5’-CGTTCACCGGCATCAACGTTT-3’	1 cycle at 94°C for 3 m; 35 cycles of 94°C for 30 s, 60.8°C for 30 s, 72°C for 30 s; 1 cycle of 72°C for 10 m	Cre Tg positive: 232bp Cre Tg negative: no product
mChat-Cre Tg PCR	Forward: 5’-AGAGTACACTGTGGGCAGGA-3’ Reverse: 5’-GCAAACGGACAGAAGCATTT-3’	1 cycle at 95°C for 5 m; 35 cycles of 94°C for 30 s, 63°C for 30 s, 72°C for 1 m; 1 cycle of 72°C for 10 m	mChat-Cre Tg positive: 350bp mChat-Cre negative: no product
ZsGreen1 PCR	Forward: 5’-GTGTACAAGGCCAAGTCCGT-3’ Reverse: 5’-CCACTTCTGGTTCTTGGCGT-3’	1 cycle at 95°C for 3 m; 35 cycles of 94°C for 20 s, 66°C for 25 s, 72°C for 30 s; 1 cycle of 72°C for 10 m	Zsgreen Tg positive: 102bp Zsgreen Tg negative: no product
Sex determination: X Chromosome	Forward: 5'-GTGAAGGAGGAATTAGGTGG-3' Reverse: 5'-GATGTGGTAATTGTCATCAC-3'	1 cycle at 94°C for 5 m; 35 cycles of 94°C for 1 m, 61°C for 1 m, 72°C for 1 m; 1 cycle of 72°C for 7 m	X Chromosome: ~1100bp
Sex determination: Y Chromosome	Forward: 5'-GTAGGTTGTTGTCCCATTGC-3' Reverse: 5'-GAGAGAGGCACAAGTTGGC-3'	1 cycle at 94°C for 5 m55; 35 cycles of 94°C for 1 m, 61°C for 1 m, 72°C for 1 m; 1 cycle of 72°C for 7 m	Y Chromosome: 272bp

Tg: transgene; bp: base pairs; m: minutes; s: seconds

Supplemental Figure 1 (S1).

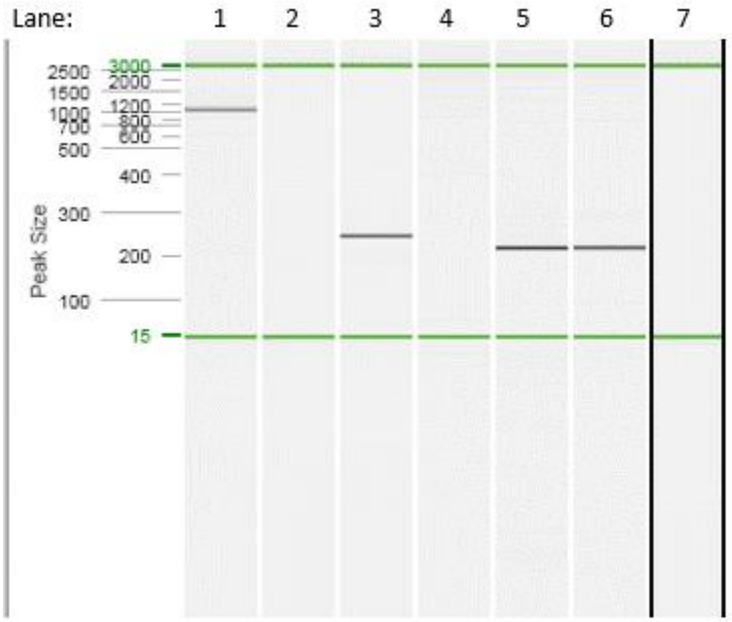

Supplemental Figure 1. Original data image used to produce Figure 1B. The samples were analyzed using a QIAxcel Advanced capillary electrophoresis system and QIAxcel ScreenGel 1.1.0 software. Lanes 1-3 are results for samples analyzed using the ZsGreen PCR assay and Lanes 4-6 are results for samples analyzed using the Cre PCR assay. Lane 7 is an empty lane. Lanes 1 & 4: animal positive for only the ZsGreen transgene; Lanes 2 & 5: animal positive for only the Cre transgene; Lanes 3 & 6: animal that is positive for both the ZsGreen transgene and the Cre transgene. The smaller amplicon detected in the ZsGreen PCR assay is due to a successful Cre-mediated recombination event that has removed the STOP cassette from the ZsGreen transgene. The molecular sizes in base pairs are indicated on the left of the image. The bands at 15 bp and 3000 bp correspond to the QX alignment markers. Lanes 1-3

are shown in the upper panel of Figure 1B and Lanes 4-6 are shown in the lower panel of Figure 1B.

Supplemental Figure 2 (S2).

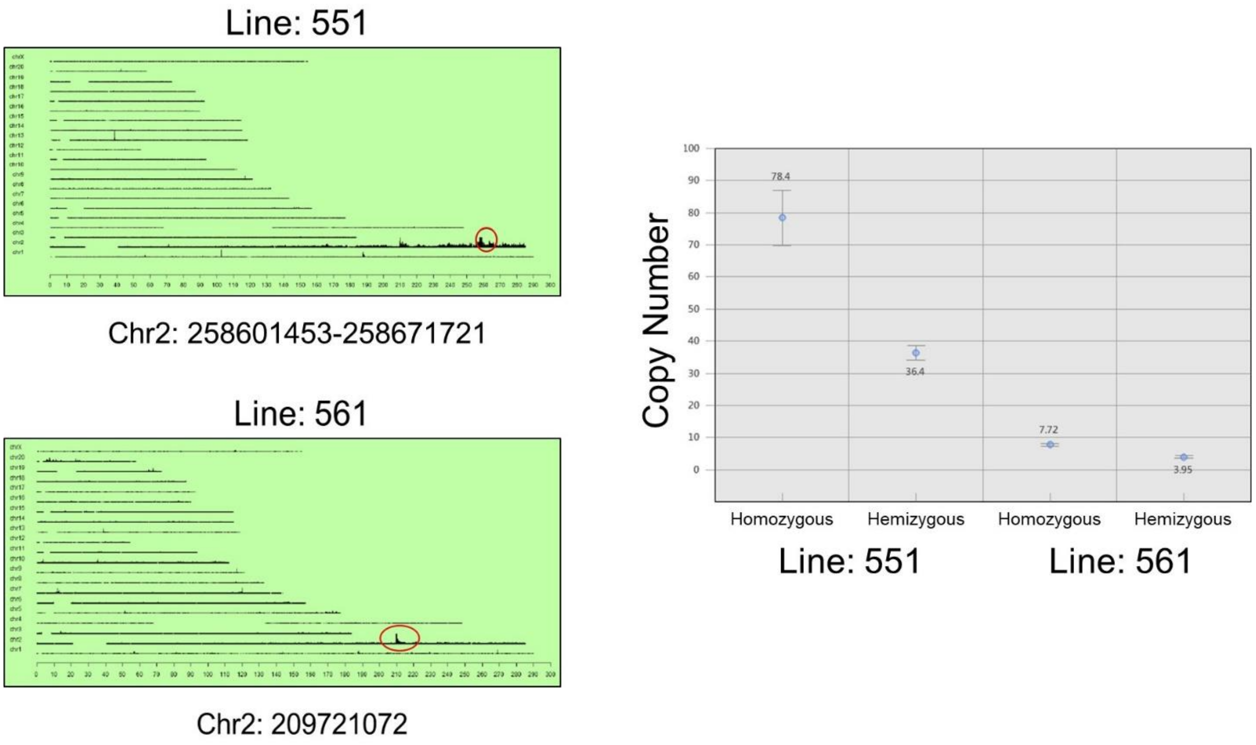

Supplemental Figure 2. Examples of TLA sequence coverage across the rat genome for F344-Tg(CAG-*lox*P-STOP-*lox*P-ZsGreen)551Bryd (Line 551) and F344-Tg(CAG-*lox*P-STOP-*lox*P-ZsGreen)561Bryd (Line 561) are shown on the left. The transgene integrated into regions of Chromosome 2 for both strains. The graph on the right shows the transgene copy number estimates for hemizygous and homozygous animals of each strain based on droplet digital PCR analysis using the BioRad QuantaSoft Analysis Pro software with Poisson error bars and a confidence interval of 95%.
